# Plasma neutrophil gelatinase-associated lipocalin is independently associated with left ventricular hypertrophy and diastolic dysfunction in patients with chronic kidney disease

**DOI:** 10.1371/journal.pone.0205848

**Published:** 2018-10-16

**Authors:** Il Young Kim, June Hyun Kim, Min Jeong Kim, Dong Won Lee, Cheol Gu Hwang, Miyeun Han, Harin Rhee, Sang Heon Song, Eun Young Seong, Soo Bong Lee

**Affiliations:** 1 Department of Internal Medicine, Pusan National University School of Medicine, Yangsan, Republic of Korea; 2 Research Institute for Convergence of Biomedical Science and Technology, Pusan National University Yangsan Hospital, Yangsan, Republic of Korea; 3 Medical Research Institute, Pusan National University Hospital, Busan, Republic of Korea; International University of Health and Welfare, School of Medicine, JAPAN

## Abstract

**Background:**

Cardiovascular disease (CVD) is a leading cause of death in patients with chronic kidney disease (CKD). Left ventricular hypertrophy (LVH) and left ventricular diastolic dysfunction (LVDD) are known as predictors of CVD in these patients. Neutrophil gelatinase-associated lipocalin (NGAL) is a biomarker of acute kidney injury. Recently, elevated NGAL levels have been reported in patients with CVD. This study aimed to evaluate the association between plasma NGAL levels and LVH/LVDD in patients with CKD.

**Methods:**

This study included 332 patients with pre-dialysis CKD (estimated glomerular filtration rate (eGFR) < 60 ml/min/1.73m^2^). Two-dimensional echocardiography was performed to measure the left ventricular mass index (LVMI). Tissue Doppler imaging was used to measure early mitral inflow velocity (E) and the peak early mitral annular velocity (E'). Diastolic function was estimated using E' and the ratio of E to E' (E/E'). The associations of echocardiographic index with clinical and laboratory variables (age, sex, diabetes, hypertension, eGFR, albumin, uric acid, calcium, phosphate, total cholesterol, hemoglobin, C-reactive protein, intact parathyroid hormone (PTH), the inferior vena cava collapse index (IVCCI) < 50%, and plasma NGAL) were investigated using univariate and multivariate analyses.

**Results:**

In multivariate logistic regression analysis, plasma NGAL was an independent predictor of LVH (OR: 1.02, 95% CI: 1.01–1.02), P < 0.001). In addition, hypertension, intact PTH, and IVCCI < 50% were independent predictors of LVH. Plasma NGAL (OR: 1.02, 95% CI: 1.01–1.02, P < 0.001) was also an independent factor of LVDD. Furthermore, hypertension, intact PTH, and IVCCI < 50% were independent predictors of LVDD. Receiver operating characteristic curve analysis (area under the curve: 0.835, 95% CI: 0.792–0.879) showed the best cutoff value of plasma NGAL for identifying LVDD was ≥ 258 ng/ml with an associated sensitivity of 77.6% and a specificity of 87.6%.

**Conclusion:**

Plasma NGAL levels were independent predictors of LVH and LVDD in patients with pre-dialysis CKD, suggesting that plasma NGAL could be a biomarker for LVH and LVDD in these patients.

## Introduction

Cardiovascular disease (CVD) is a leading cause of death in patients with chronic kidney disease (CKD) [1]. Not only do traditional risk factors contribute to the development of CVD in these patients, CKD-related risk factors such as sodium and fluid retention, anemia, inflammation, hyperparathyroidism, and uremic toxins may also be implicated [2]. In addition, structural and functional abnormalities of the heart are frequently observed in patients with CKD. Left ventricular hypertrophy (LVH) and left ventricular diastolic dysfunction (LVDD) are reported to be frequent among CKD patients [3, 4], and are associated with CVD-related mortality [5, 6]. Accordingly, the assessment of predictors for LVH and LVDD is important in CVD risk stratification.

Neutrophil gelatinase-associated lipocalin (NGAL) is a protein belonging to the lipocalin family, which is rapidly released in response to a variety of cellular stresses, including ischemia and inflammation [7]. NGAL is also known to be a promising biomarker for early diagnosis of acute kidney injury [8]. Beyond the kidney, a recent study has demonstrated that systemic and myocardial NGAL expression were upregulated in clinical and experimental heart failure [9]. Other studies have reported that systemic NGAL levels are elevated in various cardiovascular conditions, including heart failure, coronary heart disease (CHD), and stroke [10].

Several studies have reported on the associations between NGAL and cardiac structure or function in patients with chronic heart failure (CHF) [9, 11, 12]. However, the significance of these associations in CKD population is not well known. In this study, taking into account the high prevalence of LVH and LVDD in CKD patients, we hypothesized that plasma NGAL is associated with LVH and LVDD in CKD patients. To verify this hypothesis, we conducted a cross-sectional study to explore the association plasma NGAL and LVH/LVDD in pre-dialysis CKD patients with preserved left ventricular (LV) systolic function.

## Materials and methods

### Study population

Patients who had visited the nephrology clinic in Pusan National University Yangsan Hospital between 2010 and 2016 were investigated retrospectively in this cross-sectional study. Estimated glomerular filtration rate (eGFR) was determined using the Modification of Diet in Renal Disease (MDRD) equation [13]: 186 × sCr^−1.154^ × age^−0.203^ × 0.742 (if female) or × 1.21 (if African-American). All adult patients (≥ 18 years old) with CKD [eGFR < 60 ml/min/1.73m^2^] and who were not on dialysis were included. To exclude patients with acute kidney injury and to clearly identify patients with CKD, only patients whose previous serum creatinine level was known from medical records or who were followed for at least 3 months were included. Patients with valvular heart disease, congenital heart disease, cardiomyopathy, evidence of systolic heart failure [left ventricular ejection fraction (LVEF) < 50%], known CHD (previous myocardial infarction, or revascularization), and atrial fibrillation were excluded. This study protocol was approved by the Institutional Review Board of Pusan National University Yangsan Hospital (IRB No. 05-2017-178). Informed consent was waived by the IRB due to the retrospective nature of the analysis using information contained in medical charts and records, which were anonymized.

### Study variables

Demographic and clinical data on age, gender, and presence of diabetes or hypertension were obtained by reviewing medical records. Diabetes was defined as the use of diabetes medication or fasting plasma glucose concentration ≥ 126 mg/dl. Hypertension was defined as the use of hypertension medication or systolic blood pressure > 140 mmHg or diastolic blood pressure > 90 mmHg. Body mass index (BMI) was calculated by measuring the weight and height of each patient and is expressed as kg/m^2^. All blood variables including albumin, uric acid, calcium, phosphate, total cholesterol, hemoglobin, C-reactive protein (CRP), intact parathyroid hormone (PTH), and plasma NGAL were measured concomitantly. Plasma NGAL level was measured using Triage NGAL immunoassay (Alere Inc., San Diego, CA, USA) with a measurable range from 15 ng/ml to 1300 ng/ml. Intact PTH levels were measured using electrochemiluminescent immunoassay (Roche Diagnostics, Mannheim, Germany) (reference range: 10–65 pg/ml).

### Echocardiography

Transthoracic echocardiography was performed using an IE33 echo system (Philips, Amsterdam, Netherlands). All echocardiographic data were measured according to the guideline of the American Society of Echocardiography [14]. Standard echocardiographic 2-D and M-mode measurements were obtained to determine the LV mass. The LV mass was estimated using the cube formula at end-diastole (LV mass = 0.8 x [1.04 x {interventricular septum thickness + LV internal diameter + posterior wall thickness}^3^ –{LV internal diameter}^3^] + 0.6 g). LV mass index (LVMI) was calculated by dividing LV mass with the patient’s body surface area (BSA) [LVMI = LV mass (g)/BSA (m^2^)] [14]. LVH was defined as the LVMI > 115 g/m^2^ in men and > 95 g/m^2^ in women [14]. The LVEF, indicating the LV systolic function, was calculated using the biplane Simpson’s method. LVDD was assessed using both Doppler echocardiography and tissue Doppler imaging. Early mitral inflow velocity (E) and late mitral inflow velocity (A) were measured using Doppler echocardiography. Peak early mitral annular velocity (E') was computed as the average of velocities obtained at the medial and lateral annuli using tissue Doppler. The ratio of E to E' (E/E' ratio) was calculated and used to estimate the LV filling pressure. The severity of LVDD was assessed by the E' and E/E' ratio. The presence of LVDD was defined as when E' < 8 cm/s [14]. The inferior vena cava (IVC) diameter was also measured. The proportion that the IVC collapses with respiration was defined as the inferior vena cava collapse index (IVCCI). The IVCCI, which is a widely-used parameter in IVC assessment of intravascular volume, was determined as the difference between the maximum and minimum IVC diameters divided by the maximum IVC diameter [(IVCmax-IVCmin)/IVCmax] x 100% [15]. Cyclic changes intrathoracic pressure may result in the collapse of the IVC diameter of approximately 50% [15]. Thus, the IVCCI < 50% was defined as the presence of volume overload in this study.

### Statistical analysis

Continuous variables are expressed as the mean ± standard deviation and categorical variables are presented as percentages. Differences among groups were tested with one-way ANOVA for continuous variables and with the chi-square test for categorical data. Pearson’s correlation was used to investigate the correlation associated with the LVMI, E', and E/E' ratio in the study subjects. Uni- and multivariate logistic regression analyses were performed to calculate the odds ratio (OR) with 95% confidential interval (CI) for having the LVH and LVDD in the study subjects. Receiver operating characteristic (ROC) curve analysis was employed to assess the best cut-off value of plasma NGAL for predicting the LVH and LVDD in the study subjects. Values of *P* < 0.05 were considered statistically significant. All analyses were performed using the SPSS version 21.0 statistical package (SPSS, Inc., Chicago, IL, USA) and MedCalc Statistical Software version 15.8 (MedCalc Software, Ostend, Belgium).

## Results

### Baseline characteristics of study population

The baseline characteristics of the study population are shown in Table 1. Of the 332 patients, 198 were in CKD stage 3, 84 in CKD stage 4, and 50 in CKD stage 5. The mean eGFRs (ml/min/1.73m^2^) were 42.7 ± 7.9 in CKD stage 3, 21.7 ± 4.1 in CKD stage 4, and 10.0 ± 2.5 in CKD stage 5. There were no significant differences across the three groups in sex, total cholesterol, BMI, and prevalence of DM, hypertension and glomerulonephritis. Patients with advanced CKD were more likely to be old and have elevated levels of serum phosphate (P < 0.001), uric acid (P = 0.004), CRP (P < 0.001), and intact PTH (P < 0.001), whereas they were likely to have decreased levels of serum albumin (P < 0.001), calcium (P < 0.001), and hemoglobin (P < 0.001). Patients with advanced CKD were also more likely to have volume overload as indicated by IVCCI < 50% (P = 0.048). Plasma NGAL levels were significantly higher in the advanced CKD stages (P < 0.001). In the echocardiographic parameters, patients with more advanced CKD stages had a higher LVMI (P < 0.001) and prevalence of LVH (27.9% in CKD stage 3, 47.1% in CKD stage 4, and 66.0% in CKD stage 5, P < 0.001). The degree of LVDD was more severe with increasing CKD stage, which was evidenced by lower E' (P = 0.003) and higher E/E' ratio (P = 0.041). However, the prevalence of LVDD was not different across the CKD stages. There were no significant differences between the three CKD groups in LVEF.

**Table 1:** Baseline characteristics of the study population (n = 332).

	CKD stage 3 (n = 198)	CKD stage 4 (n = 84)	CKD stage 5 (n = 50)	P
Age (years)	55.3 ± 10.5	59.5 ± 11.0	63.1 ± 12.3	<0.001
Sex, male	104 (52.8%)	45 (52.9%)	27 (54.0%)	0.988
Diabetes	108 (54.8%)	45 (52.9%)	27 (54.0%)	0.958
Hypertension	134 (68.0%)	64 (75.3%)	41 (82.0%)	0.106
Glomerulonephritis	38 (19.3%)	18 (21.2%)	10 (20.0%)	0.936
History of cardiovascular disease	13 (11.7%)	16 (18.8%)	13 (26.0%)	0.029
eGFR (ml/min/1.73m^2^)	42.8 ± 7.4	21.7 ± 4.0	9.8 ± 2.2	<0.001
Albumin (g/dl)	4.2 ± 0.4	4.0 ± 0.4	3.9 ± 0.5	<0.001
Uric acid (mg/dl)	6.3 ± 1.9	6.6 ± 1.9	7.3 ± 1.8	0.004
Calcium (mg/dl)	9.2 ± 0.3	9.0 ± 0.3	8.9 ± 0.3	<0.001
Phosphate (mg/dl)	3.4 ± 0.5	3.9 ± 0.7	4.6 ± 0.9	<0.001
Total cholesterol (mg/dl)	214.3 ± 45.7	209.6 ± 46.9	216.5 ± 45.8	0.645
Body mass index (kg/m^2^)	23.9 ± 2.7	24.0 ± 2.7	23.7 ± 2.4	0.890
Hemoglobin (g/dl)	12.8 ± 1.9	11.0 ± 1.9	9.6 ± 1.2	<0.001
CRP (mg/l)	1.3 ± 1.1	1.9 ± 1.3	2.7 ± 1.4	<0.001
Intact PTH (pg/ml)	50.2 ± 20.0	112.6 ± 76.6	224.7 ± 83.7	<0.001
Plasma NGAL (ng/ml)	214.0 ± 92.4	257.7 ± 86.2	347.3 ± 159.7	<0.001
LVMI (g/m^2^)	94.6 ± 23.6	103.9 ± 25.4	119.8 ± 21.1	<0.001
E (cm/s)	62.9 ± 8.6	64.0 ± 8.3	60.8 ± 11.2	0.130
E' (cm/s)	8.0 ± 1.4	7.5 ± 1.0	7.4 ± 1.3	0.002
E/E'	8.1 ± 1.7	8.6 ± 0.7	8.6 ± 2.5	0.041
LVEF (%)	62.8 ± 8.2	61.4 ± 7.7	59.8 ± 7.60	0.053
LVH	55 (27.9%)	40 (47.1%)	33 (66.0%)	<0.001
LVDD	123 (62.4%)	54 (63.5%)	33 (66.0%)	0.895
IVCCI < 50%	16 (8.1%)	11 (12.9%)	10 (20.0%)	0.048

Data are mean ± standard deviation or (n, %). CRP, C-reactive protein; E, early mitral inflow velocity; E', peak early mitral annular velocity; eGFR, estimated glomerular filtration rate; IVCCI, inferior vena cava collapsing index = ([IVCmax-IVCmin]/IVCmax) x 100%; PTH, parathyroid hormone; LVEF, left ventricular ejection fraction; LVDD, left ventricular diastolic dysfunction; LVH, left ventricular hypertrophy; LVMI, left ventricular mass index; NGAL, neutrophil gelatinase-associated lipocalin

**Fig 1:**
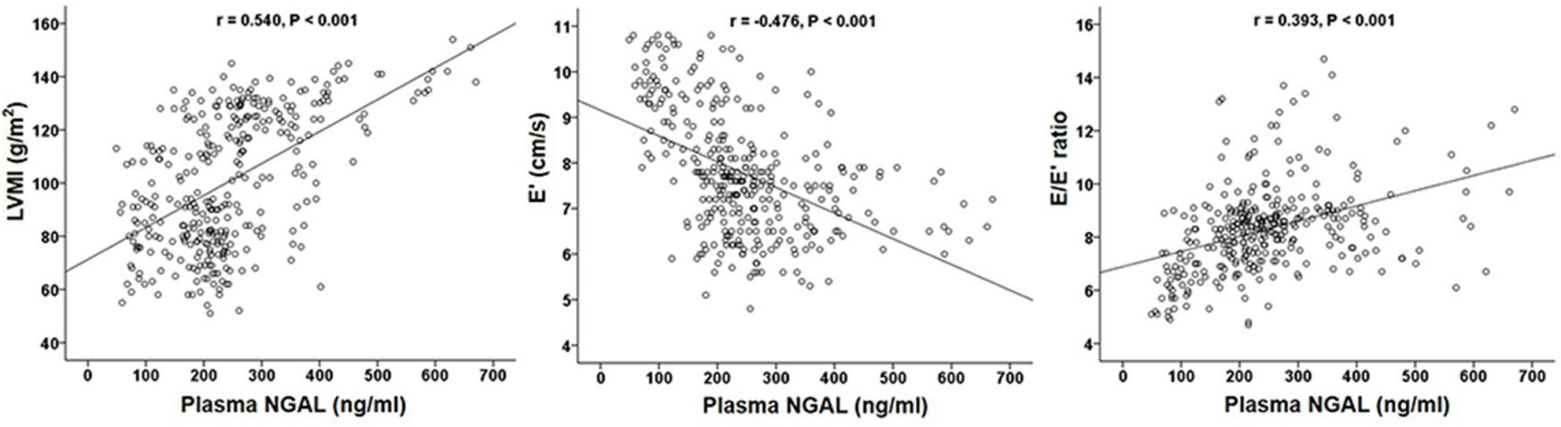
Correlations between plasma NGAL and LVMI, E', and E/E' in pre-dialysis CKD patients (n = 332). E, early mitral inflow velocity; E', peak early mitral annular velocity; LVMI, left ventricular mass index.

The mean interval from the plasma NGAL measurement to echocardiography was 31.4 ± 15.0 hours (range: 3–74 hours). Data of LVH and LVDD according to tertiles of plasma NGAL values are shown in Table 2. Patients with higher plasma NGAL levels were more likely to have higher LVMI and E/E' value and lower E'. They were also more likely to have the higher prevalence of LVH and LVDD. Correlation of plasma NGAL with the LVMI, the E', and the E/E' ratio was depicted in the form of scatterplots in Fig 1. Plasma NGAL levels were positively correlated with LVMI (*r* = 0.540, P < 0.001) and E/E' (*r* = 0.393, P < 0.001). Plasma NGAL levels were negatively correlated with E' (*r* = -0.476, P < 0.001). There was a significant correlation between LVH and LVDD. The LVMI was negatively correlated with the E (*r* = -0.363, P < 0.001) and positively correlated with the E/E' (*r* = 0.290, P < 0.001).

**Table 2:** Data of LVH and LVDD according to tertiles of plasma NGAL values (n = 332).

	Tertile 1 (<196.8)	Tertile 2 (196.8–263.0)	Tertile 3 (>263.0)	P
LVMI (g/m^2^)	89.1 ± 19.8	94.3 ± 24.3	119.1 ± 20.7	<0.001
E' (cm/s)	8.7 ± 1.4	7.5 ± 1.1	7.1 ± 1.0	<0.001
E/E'	7.5 ± 1.5	8.3 ± 1.3	9.1 ± 1.7	<0.001
LVH	11 (10.0%)	33 (29.5%)	84 (76.4%)	<0.001
LVDD	33 (30.0%)	81 (72.3%)	96 (87.3%)	<0.001

Data are mean ± standard deviation or (n, %). E, early mitral inflow velocity; E', peak early mitral annular velocity; LVDD, left ventricular diastolic dysfunction; LVH, left ventricular hypertrophy; LVMI, left ventricular mass index; NGAL, neutrophil gelatinase-associated lipocalin

### Association between plasma NGAL and LVH

Table 3 shows the variables found to be significantly associated with the presence of LVH. In univariate logistic regression analysis, age (OR: 1.06, 95% CI: 1.04–1.09, P < 0.001), hypertension (OR: 5.71, 95% CI: 3.01–10.81, P < 0.001), e GFR (OR: 0.95, 95% CI: 0.93–0.97, P < 0.001), albumin (OR: 0.52, 95% CI: 0.30–0.90, P = 0.021), phosphate (OR: 2.25, 95% CI: 1.63–3.11, P < 0.001), hemoglobin (OR: 0.85, 95% CI: 0.76–0.94, P < 0.001), intact PTH (OR: 1.02, 95% CI: 1.01–1.02, P < 0.001), plasma NGAL (OR:1.02, 95% CI: 1.01–1.02, P < 0.001), and IVCCI < 50% (OR: 5.19, 95% CI: 2.42–11.14, P < 0.001) were significant predictors of LVH. In multivariate logistic regression analysis, plasma NGAL was an independent predictor of LVH (OR: 1.02, 95% CI: 1.01–1.02), P < 0.001). In addition, age (OR: 1.05, 95% CI: 1.01–1.08, P = 0.005), hypertension (OR: 4.57, 95% CI: 1.83–11.41, P = 0.001), intact PTH (OR: 1.01, 95% CI: 1.01–1.02, P = 0.001), and IVCCI < 50% (OR: 4.10, 95% CI: 1.39–12.08, P = 0.011) were independent predictors of LVH.

**Table 3:** Univariate and multivariate logistic regression analyses for variables associated with the presence of left ventricular hypertrophy in the study population (n = 332).

	Univariate		Multivariate	
	Odds ratio (95% CI)	P	Odds ratio (95% CI)	P
Age (per 1 year increase)	1.06 (1.04–1.09)	<0.001	1.05 (1.01–1.08)	0.005
Male (vs female)	0.96 (0.62–1.49)	0.847	1.27 (0.66–2.44)	0.474
Diabetes (vs no diabetes)	0.98 (0.63–1.53)	0.854	1.13 (0.56–2.31)	0.732
Hypertension (vs no hypertension)	5.71 (3.01–10.81)	<0.001	4.57 (1.83–11.41)	0.001
History of CVD (vs no history of CVD)	1.59 (0.88–2.89)	0.127	0.69 (0.26–1.80)	0.444
eGFR (per 1 ml/min/1.73m^2^ increase)	0.95 (0.93–0.97)	<0.001	1.00 (0.97–1.04)	0.940
Albumin (per 1 g/dl increase)	0.52 (0.30–0.90)	0.021	1.17 (0.47–2.93)	0.732
Uric acid (per 1 mg/dl increase)	1.05 (0.93–1.18)	0.426	1.03 (0.86–1.22)	0.766
Calcium (per 1 mg/dl increase)	0.81 (0.43–1.56)	0.534	2.25 (0.84–6.07)	0.108
Phosphate (per 1 mg/dl increase)	2.25 (1.63–3.11)	<0.001	1.39 (0.80–2.39)	0.234
Total cholesterol (1 per 1 mg/dl increase)	1.00 (1.00–1.01)	0.999	0.99 (0.99–1.00)	0.128
Body mass index (per 1 kg/m^2^ increase)	1.01 (0.93–1.10)	0.788	0.94 (0.83–1.07)	0.346
Hemoglobin (per 1 g/dl increase)	0.85 (0.76–0.94)	0.002	1.08 (0.90–1.30)	0.406
CRP (per 1 mg/l increase)	1.14 (0.97–1.35)	0.121	0.83 (0.63–1.10)	0.205
Intact PTH (per 1 pg/ml increase)	1.02 (1.01–1.02)	<0.001	1.01 (1.00–1.02)	0.001
Plasma NGAL (per 1 ng/ml increase)	1.02 (1.01–1.02)	<0.001	1.02 (1.01–1.02)	<0.001
IVCCI < 50% (vs IVCCI ≥ 50%)	5.19 (2.42–11.14)	<0.001	4.10 (1.39–12.08)	0.011

CI, confidential interval; CRP, C-reactive protein; CVD, cardiovascular disease; eGFR, estimated glomerular filtration rate; IVCCI, inferior vena cava collapsing index = ([IVCmax-IVCmin]/IVCmax) x 100%; PTH, parathyroid hormone; LVMI, left ventricular mass index; NGAL, neutrophil gelatinase-associated lipocalin

### Association between plasma NGAL and LVDD

Table 4 shows the variables found to be associated with the presence of LVDD in the study population. In univariate logistic regression analysis, hypertension (OR: 4.85, 95% CI: 2.90–8.08, P < 0.001), history of CVD (OR: 2.16, 95% CI: 1.08–4.29, P = 0.029), intact PTH (OR: 1.01, 95% CI: 1.00–1.01, P < 0.001), plasma NGAL (OR: 1.02, 95% CI: 1.01–1.02, P < 0.001), and the IVCCI < 50% (OR: 12.0, 95% CI: 2.83–50.84, P = 0.001) were the significant predictors of LVDD. In multivariate logistic regression analysis, plasma NGAL (OR: 1.02, 95% CI: 1.01–1.02, P < 0.001) was an independent factor of LVDD. Furthermore, hypertension (OR: 3.23, 95% CI: 1.61–6.48, P = 0.001), intact PTH (OR: 1.02, 95% CI: 1.01–1.02, P = 0.027), and IVCCI < 50% (OR: 9.22, 95% CI: 1.91–44.49, P = 0.006) were independent predictors of LVDD.

**Table 4:** Univariate and multivariate logistic regression analyses for variables associated with the presence of left ventricular diastolic dysfunction in the study population (n = 332).

	Univariate		Multivariate	
	Odds ratio (95% CI)	P	Odds ratio (95% CI)	P
Age (per 1 year increase)	1.01 (0.99–1.03)	0.217	0.98 (0.95–1.00)	0.172
Male (vs female)	1.04 (0.66–1.62)	0.878	1.21 (0.66–2.22)	0.534
Diabetes (vs no diabetes)	1.24 (0.80–1.94)	0.344	1.23 (0.65–2.35)	0.528
Hypertension (vs no hypertension)	4.85 (2.90–8.08)	<0.001	3.23 (1.61–6.48)	0.001
History of CVD (vs no history of CVD)	2.16 (1.08–4.29)	0.029	1.10 (0.42–2.88)	0.849
eGFR (per 1 ml/min/1.73m^2^ increase)	0.99 (0.98–1.00)	0.259	1.03 (1.00–1.06)	0.104
Albumin (per 1 g/dl increase)	0.71 (0.40–1.24)	0.230	0.94 (0.40–2.18)	0.876
Uric acid (per 1 mg/dl increase)	1.02 (0.90–1.14)	0.771	1.13 (0.96–1.33)	0.131
Calcium (per 1 mg/dl increase)	1.38 (0.72–2.66)	0.335	1.69 (0.66–4.31)	0.275
Phosphate (per 1 mg/dl increase)	1.08 (0.80–1.45)	0.632	0.73 (0.43–1.23)	0.239
Total cholesterol (1 per 1 mg/dl increase)	1.00 (1.00–1.01)	0.274	1.00 (0.99–1.00)	0.750
Body mass index (per 1 kg/m^2^ increase)	1.02 (0.93–1.10)	0.727	0.96 (0.85–1.08)	0.494
Hemoglobin (per 1 g/dl increase)	0.98 (0.87–1.09)	0.730	1.10 (0.93–1.30)	0.284
CRP (per 1 mg/l increase)	0.94 (0.80–1.12)	0.493	0.81 (0.62–1.04)	0.101
Intact PTH (per 1 pg/ml increase)	1.01 (1.00–1.01)	<0.001	1.01 (1.00–1.01)	0.027
Plasma NGAL (per 1 ng/ml increase)	1.02 (1.01–1.02)	<0.001	1.02 (1.01–1.02)	<0.001
IVCCI < 50% (vs IVCCI ≥ 50%)	12.0 (2.83–50.84)	0.001	9.22 (1.91–44.49)	0.006

CI, confidential interval; CRP, C-reactive protein; CVD, cardiovascular disease; eGFR, estimated glomerular filtration rate; IVCCI, inferior vena cava collapsing index = ([IVCmax-IVCmin]/IVCmax) x 100%; PTH, parathyroid hormone; LVMI, left ventricular mass index; NGAL, neutrophil gelatinase-associated lipocalin

### Performance of plasma NGAL for predicting the LVH and LVDD

**Fig 2:**
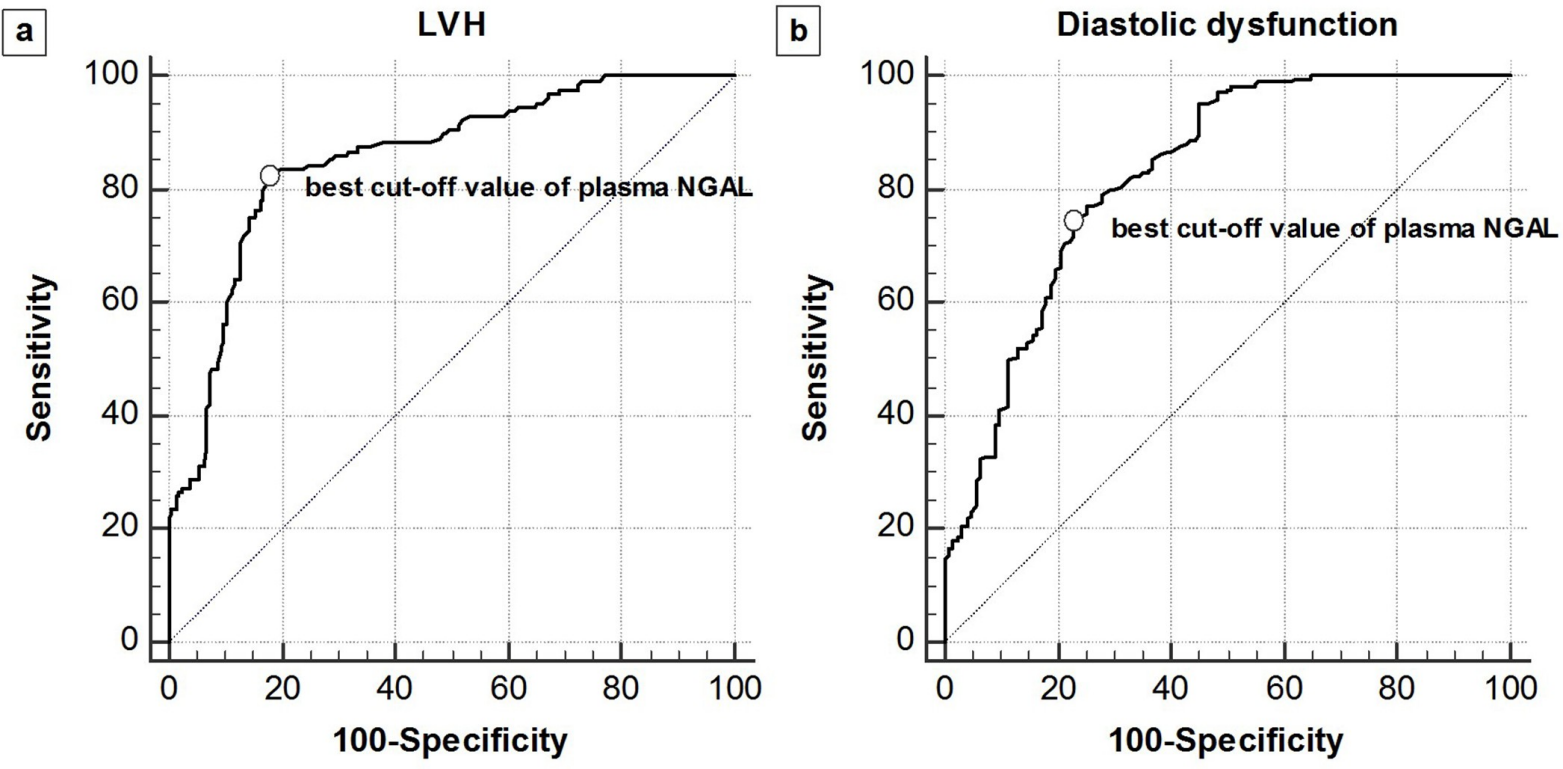
**ROC curves of plasma NGAL for predicting the presence of LVH (a) and LVDD (b) in patients with pre-dialysis CKD (n = 322)**. The AUCs of plasma NGAL were 0.855 (95% CI, 0.813–0.819) for LVH and 0.827 (95% CI, 0.782–0.866) for LVDD, respectively. The best cut-off value of plasma NGAL for predicting the presence of LVH was > 243 ng/ml with the associated sensitivity of 82.0% (95% CI, 74.3–88.3%) and specificity of 82.4% (95% CI, 76.4–87.3%). The best cut-off value of plasma NGAL for predicting the presence of LVDD was > 215 ng/ml with the associated sensitivity of 74.8% (95% CI, 68.3–80.5%) and specificity of 77.0% (68.6–84.2%).

To investigate the diagnostic power of plasma NGAL for predicting the presence of LVH and LVDD in patients with pre-dialysis CKD, ROC analysis was performed (Fig 2). The area under the curve (AUCs) of plasma NGAL were 0.855 (95% CI, 0.813–0.819) for LVH and 0.827 (95% CI, 0.782–0.866) for LVDD. The best cut-off value of plasma NGAL for predicting the presence of LVH was > 243 ng/ml with the associated sensitivity of 82.0% (95% CI, 74.3–88.3%) and specificity of 82.4% (95% CI, 76.4–87.3%). The best cut-off value of plasma NGAL for predicting the presence of LVDD was > 215 ng/ml with the associated sensitivity of 74.8% (95% CI, 68.3–80.5%) and specificity of 77.0% (68.6–84.2%).

## Discussion

LVH and LVDD are common and their prevalence and severity are known to increase with the progressive decline of renal function in CKD patients [3, 4, 16]. LVH is an adaptive response to pressure and volume overload. However, sustained overload in combination with uremia-associated factors such as anemia and hyperparathyroidism lead progressively to a maladaptive hypertrophic response, resulting in LVDD [17]. In this study, the LVMI and prevalence of LVH increased with advanced CKD stages. The patients with a higher CKD stage also showed a more pronounced LVDD as demonstrated by lower E' and higher E/E'. The prevalence of LVDD tends to be higher with increasing CKD stages although it was not statistically different among three CKD groups.

NGAL is upregulated and secreted in kidney injury. In CKD patients, blood NGAL level is known to be elevated and is a predictor of the progression of CKD [18]. Our study also showed that plasma NGAL levels increased as the CKD stage increased. Recent studies have reported that NGAL is associated with not only renal failure but also cardiovascular disease [10]. In patients with CHF, systemic NGAL levels correlated with the severity of CHF [New York Heart Association (NYHA) class] and N-terminal pro B-type natriuretic peptide (NT-proBNP) [9, 11, 19]. NGAL has also been reported to have the prognostic value to predict the all-cause or cardiovascular mortality in CHF patients [11, 12]. In CHD, serum NGAL levels were associated with presence and severity of CHD [20, 21]. Serum NGAL levels were elevated in patients with the acute cerebrovascular accident and lasted up to one year [22]. High levels of NGAL at the time of acute cerebrovascular event were associated with higher cardiovascular mortality [23].

With regards to the association between NGAL and cardiac structure or function, previous studies showed inconsistent results. Bolignano et al. reported that serum NGAL was correlated with LV systolic function in 46 elderly patients with CHF [11]. Yndestad et al. found serum NGAL was not correlated with echocardiographic indices of LV systolic function in 150 patients with CHF [9]. In another study, Shrestha et al. reported plasma NGAL levels were modestly associated with indices of LVDD in 130 patients with CHF, but not after adjustment for renal function [12]. All of the studies above [9, 11, 12] included patients with CHF. To the best of our knowledge, the present study is the first to examine the association between plasma NGAL and LVH/LVDD in CKD population.

How the plasma NGAL levels are associated with the LVH and LVDD in CKD patients is unclear. Ventricular remodeling in LVH and LVDD is a highly complex process that not only involves alterations in the cardiomyocyte but also alterations in the extracellular matrix, and matrix metalloproteinase (MMP) is an important mediator in this process [9]. NGAL forms a heterodimer complex with MMP-9, inhibiting its auto-degradation, leading to prolonged and enhanced activity of MMP-9 [24]. MMP-9 has been implicated in ventricular remodeling and cardiac dysfunction [25], thus suggesting a potential role for NGAL in the pathogenesis of LVH and LVDD. Indeed, multiple studies demonstrated the enhanced systemic and myocardial NGAL expression in clinical and experimental heart failure [10]. In this study, we are not sure whether the increased plasma NGAL observed in CKD patients has a direct harmful effect on the heart. However, we think that increased plasma NGAL may contribute to ventricular remodeling (LVH and LVDD) by enhancing the MMP-9 activity. In addition, the involvement of NGAL in myocardial inflammation and oxidative stress suggest the role of NGAL in ventricular remodeling [9].

In this study, patients with advanced CKD were more likely to have higher plasma NGAL levels. This result suggests that chronic renal injury might enhance the production or reduce the clearance of NGAL [26]. The previous studies suggested that elevated NGAL level is not just the result of decreased clearance. Mori and Nakao suggested that the elevated NGAL in CKD is the consequence of a sustained production by inflamed but vital tubular cells [27]. The higher plasma NGAL levels with increasing CKD stage suggests plasma NGAL might reflect the progression of inflammation in CKD. NGAL is now recognized as an inflammation marker following different types of damage. It is well known that chronic uremia is associated with inflammation induced by endothelial dysfunction, advanced atherosclerosis, immune system alteration, and oxidative stress [28]. All these conditions may be potential causes of elevated plasma NGAL level in CKD.

Our study has several limitations. First, due to the cross-sectional design of our study, it is difficult to establish temporal relationship and causality. Future prospective studies are needed to verify the potential causal relationship between plasma NGAL and the development of LVH and LVDD in CKD patients. Second, there was no investigation about the medication history at the time of study enrollment due to limited patient information. Thus, we could not rule out the possibility that antihypertensive medications such as angiotensin-converting enzyme inhibitor or angiotensin receptor blocker might have affected the association between plasma NGAL and LVH/LVDD. Third, the study subjects were highly selected. In fact, patients with normal LVEF without valvular or ischemic heart disease are not common in the setting of patients with CKD, especially advanced CKD stage. Therefore, it is unclear if our results can be extrapolated to all patients with CKD. However, we excluded patients with these confounding abnormalities to reveal the relationship between NGAL and LVH/LVDD more clearly.

Despite the limitations, our study has some clinical implications. Recently, combined cardiac and renal dysfunction has gained considerable attention. The cardio-renal syndrome (CRS) comprises a broad spectrum of diseases within which both the heart and kidneys are involved, acutely or chronically [29]. Of the 5 types of CRS, type-4 CRS, defined as the chronic reno-cardiac disease, is characterized by cardiovascular involvement in CKD patients [29]. In type-4 CRS, the BNP and NT-proBNP are reported to be a biomarker for heart failure in CKD patients reflecting myocardial injury due to hypertension, volume overload, and cardiac remodeling [30]. In this study, plasma NGAL levels were demonstrated to be independent predictors of LVH and LVDD in pre-dialysis CKD patients. Thus, as with the BNP and NT-proBNP, we suggest plasma NGAL could be a biomarker for LVH and LVDD in type-4 CRS.

In conclusion, this study demonstrates for the first time the association between plasma NGAL levels and LVH/LVDD in pre-dialysis CKD patients with preserved LV systolic function. We found that plasma NGAL levels were independently associated with LVH and LVDD in pre-dialysis CKD. We also showed the best cut-off value of plasma NGAL for predicting the presence of LVH and LVDD. Although the results of our study suggest that plasma NGAL may have a potential role as a biomarker for LVH and LVDD in pre-dialysis CKD patients, future studies are mandatory to clarify our findings.

## Supporting information

S1 File The values of baseline characteristics in the study subjects (n = 332). (XLSX)
